# Explaining motivational factors of employees’ behavior towards customers’ satisfaction using the theory of planned behavior

**DOI:** 10.1371/journal.pone.0314431

**Published:** 2024-11-22

**Authors:** Yeshwork Gizaw Zewdie, Demis Alamirew Getahun, Yitayal Alemu Mengistu, Sefinew Alemu Mekonnen, Missaye Mulatie Mengstie

**Affiliations:** 1 Department of management, College of business and economics, University of Gondar, Gondar, Ethiopia; 2 Department of special needs and inclusive education, College of education, University of Gondar, Gondar, Ethiopia; 3 Department of Veterinary Epidemiology and Public Health, College of Veterinary Medicine and Animals Sciences, University of Gondar, Gondar, Ethiopia; 4 Department of Psychology, College of Social Sciences and the Humanities, University of Gondar, Gondar, Ethiopia; University of Tartu, ESTONIA

## Abstract

Lack of customer satisfaction in public service is one of the most important problems particularly in developing countries. Despite the efforts by governments to improve public service delivery, the resulting outcomes are quite limited. This demands evidence not only on the demand side but also on the supply side of the public service delivery in that how to motivate employees to improve their performance in satisfying customers. It is believed that finishing customers’ issues within required time and serving them with impartiality are few of the many factors satisfying customers. The Theory of Planned Behavior was used to explore the intentions of employees towards finishing customers’ issues within required time and serving customers with impartiality, and to study associations between their intentions and any of three factors (attitude, subjective norm and perceived behavioral control) that, according to the Theory of Planned Behavior, determine intentions. A total of 188 questionnaires were completed by employees in 12 organizations. Majority of the employees (88.8%) had a positive intention to serve customers with impartiality; 66.5% had the intention to finish customers’ issues within required time. Employees had a more positive attitude and perceived behavioral control but lower subjective norm toward implementing both intentions. Attitude was positively associated (P<0.05) with the intention to serve customers with impartiality. Subjective norm was negatively associated with the intention to finish customers’ issues within required time. Intervention programs aiming to increase the intention of employees toward customers’ satisfaction should primarily focus on changing employees’ attitude and secondarily on improving their subjective norms.

## Introduction

Despite the importance of private sector in driving growth and development, the public service is the main agent that is responsible for creating inter-linkages between service users in an economy [1]. Public services are activities of governments’ in the public domain for the benefit of the public, such as policing and public health [2]. Efficient and effective public services delivery is now main agenda of most countries demanding continuous reform to act in the best interest of delivering high public services [3]. Lack of customers’ satisfaction in the public service sector is one of the most serious problems worldwide; particularly in most of developing countries. Because of this reason, countries do continuous public service delivery reform to fit the dynamic environment and changing customer needs [4]. However, despite the civil service reforms, the resulting outcomes have been quite limited and there are still many challenges and problems in the public service delivery [5].

Improving public service delivery demands identifying organizational and individual factors limiting customers’ satisfaction and giving solution for higher level public service performance [6]. The capacity and willingness to take action when things go wrong should be done at the individual level in transaction with the public as well as at the organizational level, in relation to the entire service delivery program. Decentralization public service provision and establishing regulatory oversight of staff who deals with customers is essential for an organization to insure adequate levels of quality service provision [7]. Individual factors such as motivating employees in such a way that it will bring about behavioral change and enhancing their performance brings higher customer satisfaction. The decision to improve performance of public service delivery demands attitudinal change and motivation of employees thereby enhancing their performance [6]. Motivation is what drives someone to behave in a certain way or to take a particular action and energizes one’s goal-oriented behavior [8]. Incentives, employee’s competence and commitment determine the attitude and performance of employees [6]. Incentives in form of good salaries, good working conditions or promotion make employees to work harder [9]. If every employee of an organization is provided with better compensation, everybody will be motivated to exhibit superior performance. The better the pay, the better is the performance [10]. However, money is not the only factor that motivates employees to improve public service delivery [11]; performance of employees is not only influenced by incentives they are subjected to. For instance, incentives that motivate some people may not motivate others. High level of performance in the public service depends also on the willingness of employees to change their behavior. Strategies that enhance civil servants’ motivation to improve public service delivery might, therefore, be an important part of customer satisfaction.

Several researches have been conducted on customer satisfaction on private sectors such as insurance, hotel, bank and the like [12] and on the public organizations [13, 14]. In the context of developing countries, the public sector is the dominant player in public service delivery [1]. Therefore, more research is needed to understand customers’ satisfaction on public service delivery. Moreover, all of the studies both in private and public organizations are performed on demand side of the public service. However, effective and efficient public service delivery depends also on the supply side in that employees may not perform well to satisfy customers. This side of the service delivery function is affected, among other things, by behavior of employees in the public service. This demands evidence about how to motivate employees in such a way that it will bring about attitudinal change and higher customer satisfaction [6]. To our knowledge, studies that investigated factors motivating employees to improve their performance are very limited. The theory of planned behavior (TPB) framework has been used in several studies to obtain insight in the psychological factors that influence intentions related to changing behavior. This approach has, for instance was used by Jardali et al. [15] to measure intentions among employees towards the use of a balanced scorecard and information system, by Liao et al. [16] to study customer satisfaction in the continued use of e-service.

The present study was aimed, first to identify determinants of motivation of employees: employees’ attitude (AT), subjective norms (SN) and perceived behavioral control (PBC) are related to their intention to perform public service toward customers’ satisfaction. A second aim was to explore socio-demographic characteristics potentially affecting employees’ AT, SN and PBC that are associated with their intentions.

## Literature review

### The public sector service

Globally, the service sector plays a great and significant role to the economies of all nations. One of the main responsibilities of the service sector is public service, which encompasses all government-related endeavors. The public sector has responsibility and accountability for delivering efficient and effective services to communities and societies as a customer [13]. For public service organizations, customer satisfaction has grown in importance [17] and is a strategic initiative to help countries reach their goals [18]. Different customers have different expectations and perceptions of performance [16]. The customer feels disappointed and unsatisfied if the performance doesn’t live up to their expectations. When the performance surpasses the client’s expectations, they are extremely happy. Thus, the joy a person experiences when a product or service fulfills his or her needs is referred to as customer satisfaction [19]. Customer satisfaction, to put it briefly, is the degree to which perceived performance meets customer expectations [20]. It is also thought of as a behavior that compares inputs received prior to and after acquisitions [21].

Numerous factors affect customer satisfaction. According to Teshager and Minota [6], the two main elements influencing customer satisfaction are individual behavior and organizational culture. An important component of an organization’s internal environment is its organizational culture, which encompasses elements such as individual beliefs, corporate values, working environment, promotion of a code of behavior, and employee guidance and control. Decentralization of public service delivery, regulatory control of customer facing employees, and corrective action when issues arise should all be implemented at the organizational level with regard to the service delivery program as a whole [22].

Customer satisfaction is entirely dependent on the individual with whom the client interacts, even while organizational culture is essential for providing high quality public services [23]. Any organization’s human resources, or people, are the most important component that must be influenced and convinced to complete duties [24]. Public sector workers have a duty to the community or its clients in order to satisfy the general public [25]. However, the ability of the service provider to satisfy the client is intimately linked to the effectiveness of the organization [26]. A service provider will have a higher probability of satisfying a customer if they are able to provide additional services like excitement, higher-quality service, or a wide range of services. Customers will be happier with the service, for example, if they can save time [27]. However, based on the majority of observations, public personnel have functioned more like masters without any sense of accountability or transparency than as servants of the people [24].

### Employee motivation

Motivated workers who can carry out their responsibilities well are needed to run the public sector. Employee motivation is influenced by both organizational and individual factors. According to Esther and Rosemarie [26], motivated workers are more likely to be dedicated, effective, creative, and have a higher level of job satisfaction. These traits can result in better organizational performance, which in turn has a favorable effect on customer satisfaction. Since employees interact directly with customers and have a significant impact on their satisfaction, employee motivation can be utilized as a tool to increase customer satisfaction [28]. Organizational performance will increase with employee motivation to complete tasks [24]. This emphasizes the necessity of having a driven public employee [26]. Yet, organizations always face a difficult task when it comes to employee motivation because it is unclear exactly what drives employees [29]. A motivating factor for one employee might not be for another. This is the reason why researchers and managers continue to try to comprehend and explain employee motivation [30].

In an overall effort to strengthen the supply side to deliver effective and efficient public services, governments are implementing a variety of institutional reforms and capacity enhancement interventions on significant service delivery agencies through public sector capacity building programs. A number of initiatives have been put into place in Ethiopia to raise the standard of service delivery mechanisms and the citizen administration relationship. For instance, the nation has implemented reforms to increase the effective and result-oriented public sector. However, there hasn’t been much actual execution or resultant outcomes [5].

### Theoretical framework

Theory of planned behavior (TPB) evolved as the Theory of reasoned action (TRA) in 1980 to predict a person’s intention to engage in a behavior. According to Fishbein and Ajzen [31] and Ajzen and Fishbein [32], the theory was designed to explain all behaviors over which people possess the capacity for self-control. With the addition of the concept of perceived behavior control (PBC), TPB is an expanded theory of TRA. The elements outside of a person’s control that contribute to the accomplishment of behavioral objectives are captured by perceived behavior control [33]. TPB claims that behavioral intention, the main factor influencing a person’s behavior, is the essential element of this model. The TPB states that a behavioral intention indexes a person’s motivation either to perform or not perform a behavior and is the immediate antecedent of an action [34].

**Fig 1:**
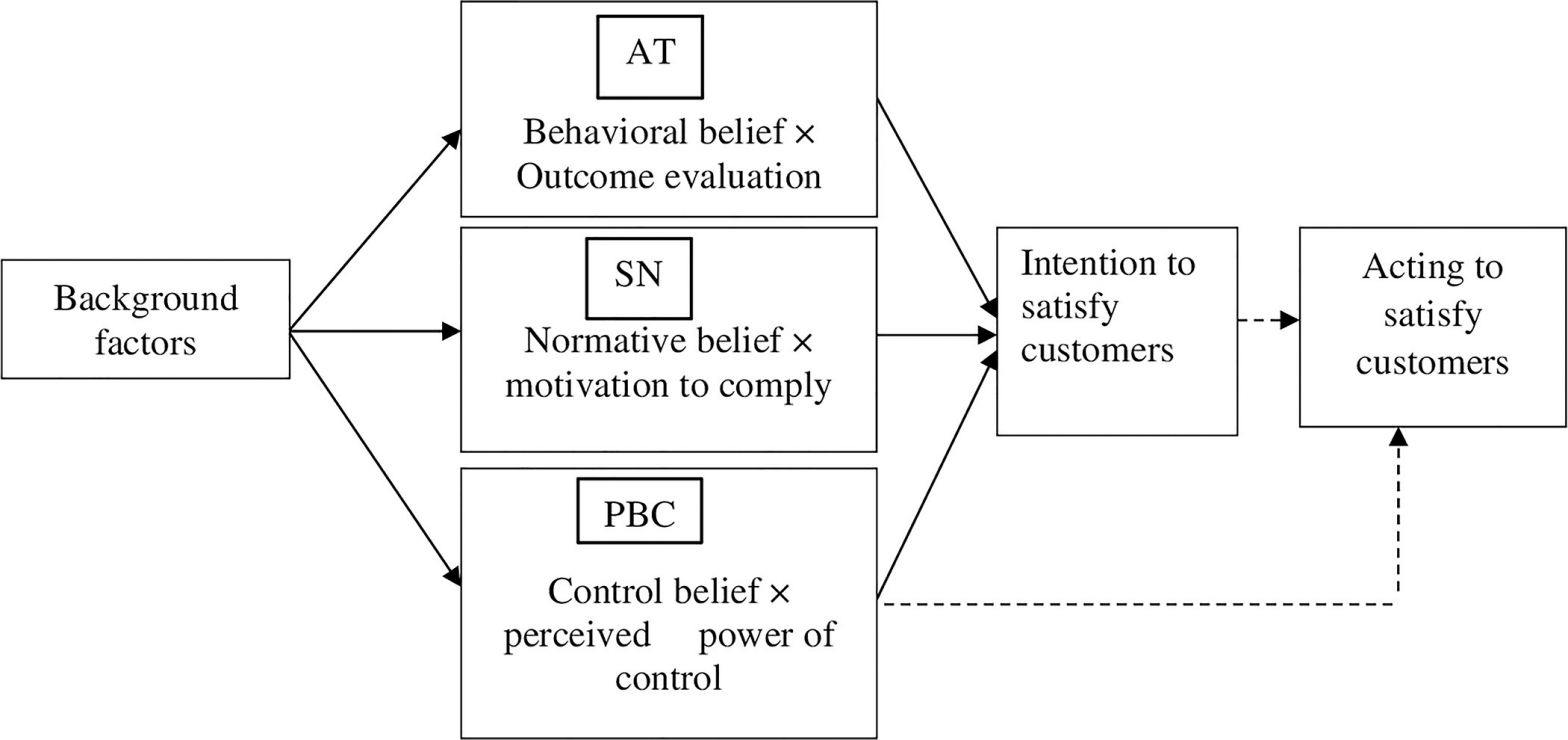
Framework of the theory of planned behavior model on the intention to participate in improving public service performance (adapted from Ajzen [35]). AT = Attitude, SN = Subjective norm, PBC = Perceived behavioral control. Dotted lines indicate associations that will not be studied.

In TPB, there are three antecedents of intention: attitudes, subjective norm, and perceived behavioral control that predict someone’s intention to perform certain behavior [35]. The theory assumes that a person’s intention either to perform or not perform behavior of different kinds can be predicted with high accuracy from persons’ AT, SN, and PBC (Fig 1) [35]. The attitude towards engaging in a behavior pertains to an individual’s perception of the personal desirability associated with that behavior [36]. A favorable or unfavorable attitude has a direct relation to the strength of the behavioral beliefs regarding the anticipated outcomes, which can be articulated through an expectancy value framework. The subjective norm encompasses an individual’s perception of social pressures that influence the decision to either engage in or refrain from the behavior [35]. This concept is linked to normative beliefs concerning the expectations of others. Perceived behavioral control arises from an individual’s conviction that they possess adequate resources, capabilities, and opportunities to execute a specific behavior. As noted by Ajzen [35], perceived behavioral control reflects an individual’s assessment of the relative ease or difficulty involved in undertaking a particular behavior.

By changing these three ‘predictors’, it is possible to increase the person’s intention to implement a desired action and thus to increase person’s motivation to actually execute that behavior [34, 37]. The variables in the TPB model are psychological (internal) constructs that can be seen as latent variables that can be approximated from response [37]. With regard to employees in the public service, AT is determined by the employees’ beliefs about the importance of performing service delivery (behavioral beliefs) and the corresponding positive or negative evaluation employees give to this effect (outcome evaluation). Subjective norms are determined from beliefs about how other people referent to employees would like them to behave (normative beliefs) and the motivation to comply with these referents (motivation to comply). Perceived behavioral control is determined by the employees’ belief whether they have the necessary conditions (control belief) including knowledge, time and labor and how confident they feel about being able to perform or not perform customers’ issues (perceived power of control) [35, 37].

## Materials and methods

### Research design

The study was conducted in a cross-sectional design in a quantitative research approach. According to Sekaran and Bougie [38], quantitative research is explaining phenomena by collecting numerical data usually to explain the nature of certain relationships or establish the differences among groups. It deals with quantifying and analyzing variables in order to get results [39, 40].

Data was collected from employees who were working in the public service and were willing to participate in the study. Respondents’ AT, SN and PBC were taken as independent variables while their intention was taken as the dependent variable. To identify socio-demographic characteristics affecting determinants of intention, TPB variables associated with intention were taken as dependent variables. Both the dependent and independent variables were measured at the same point in time using a single questionnaire.

A questionnaire was prepared in English before it was translated to local language, Amharic, and back to English to check consistency of the questions. Corrections were made to the translation based on the comparison of the different versions. However, as we have used a modified version of a validated questionnaire used in an earlier related study, we did not check construct validity or any other validity measure to our questionnaire by performing pilot interviews. We used both open and close ended questions although majority of the questions were close ended. The questionnaires contained two types of items: questions asking socio-demographic characteristics of employees, and questions that address psychological behavior of employees related to their intention to satisfy customers. These questions were designed to elicit the level of intention of employees for proposed activity and to measure the psychological factors explaining their intention, i.e. AT, SN, and PBC.

The questionnaire asked employees to complete their response to each statement using a seven point Likert scale that ranges from strongly disagree to strongly agree. The level of intention to participate in the proposed activity was assessed by different statements posed for each of the defined activity. Psychological variables were either elicited directly or derived from employees’ beliefs and their corresponding outcome evaluations [41]. This study used direct measures, similar to Thomas et al. [42]. Each of the three conceptually independent psychological models constructs measure using a specific set of rating scale statements.

Attitude was measured by statements describing an outcome related to customer satisfaction: timely service delivery (finish customers’ issues within required time) and serving customers with impartiality. In addition to these activities, specific AT statements were included to know the general attitudes towards improving performance in public service. The SN was assessed by statements asking about the importance of the opinion of groups of people in deciding to participate in improving performance in public service. In this study, groups of people: spouse, friends, boss and other employees seen as important others. Perceived behavioral control measure related to the perceived ability to execute the proposed activities in relation to availability of time and their ability. The English version of the whole questionnaire is available in supporting information file (S1 Table).

### Ethical consideration

For the questionnaire-based study that is described here, no formal approval by the Institutional Review Board of the University of Gondar was required. However, informed verbal consent was obtained after giving explanation about the aim of the study and anonymity of the data analysis before distributing the questionnaire to volunteered participants. All data were analyzed and reported anonymously.

### Sampling and data collection

We have collected a list of 1050 employees from 15 public service organizations. Considering 35% non-response rate, 290 employees were randomly selected from the list. The 290 selected employees were approached and asked for willingness to participate after telling them the aim and importance of the study, and anonymity of the data summarization and analysis. Then, a questionnaire was distributed to employees volunteered to participate in the study. Explanation was given to the volunteered employees how to complete the questionnaire and questionnaires were left for them to be completed within two days. A total of 188 employees (65% of the employees who were volunteered to participate in the study) from 12 public service organizations completed and turned the questionnaire back for re-collection.

### Socio demographic variables

Twelve socio-demographic variables deemed to be potential risk factors for TPB factors were included in the questionnaire (Table 1). Six of the variables: level of education, age of respondents, marital status, family size, salary and years of service grouped into three classes while others were classified into two levels.

**Table 1:** Socio-demographic variables potentially related to attitude (AT), subjective norms (SN) and perceived behavioral control (PBC) with respect to the intentions towards satisfying customers.

Socio-demographic factor	Level
**Sex**	Male or female
**Level of education**	Diploma, first degree or master’s degree
**Age**	≤ 30 years, 31 to 40 years or >40 years
**Marital status**	Single, couple or divorced & widow
**Family size**	≤ 3 persons, 4 to 6 persons or > 7 persons
**Having child**	No/Yes
**Salary**	≤ 7,000, > 7,000 to 10,000 or > 10,000
**Presence of income other than salary**	No/Yes
**Years of service**	< 10 years, 10 to 15 years or > 15 years
**Experience of leadership**	No/Yes
**Manager currently**	No/Yes
**Training about improving civil service delivery**	No/Yes

### Data management and analysis

Accuracy of the data was checked to minimize data entry error. Descriptive statistics such as, mean values, frequency distribution and percentages were used to summarize the data. The score given for each of the behavioral, normative and control belief statements were multiplied by the corresponding outcome evaluation, motivation to comply, or perceived power of control, respectively, to create a new variable that represented the product score. In this way, product composites were created for AT, SN and PBC. For the domains with two or more items, Cronbach’s alpha was calculated among the product composites of AT, of SN and of PBC to test internal consistency. The product composites were considered to have internal consistency and, therefore, measure similar construct, if Cronbach’s alpha was ≥0.7 [43]. In that case, product composites were averaged to obtain one single measure (mean score) for AT, SN and PBC (Eqs 1–3). For those constructs where the Cronbach’s alpha is <0.7, product composites were used as separate TPB factors.


$$AT=\frac{\sum_{i=1}^{n}(bbi*oei)}{n}$$ [1]

Where i = the product composite item i and n = number of items for AT, bb = behavioral belief, oe = outcome evaluation.


$$SN=\frac{\sum_{i=1}^{n}\left({nb}_{i}*{mc}_{i}\right)}{n}$$ [2]

Where i = the product composite item i and n = number of important referents for SN, nb = normative belief, mc = motivation to comply.


$$PBC=\frac{\sum_{i=1}^{n}\left({cb}_{i}*{ppc}_{i}\right)}{n}$$ [3]

Were i = the product composite item i and n = number of relevant control belief items, cb = control belief, ppc = perceived power of control.

The TBP factors were classified into categories based on the distribution of the average score if Cronbach’s alpha is ≥0.7, or the score of separate TPB factors if Cronbach’s alpha is <0.7 [44] as weak AT, weak SN and weak PBC (product composite ≤0); moderate AT, moderate SN and moderate PBC (>0 to <18); and strongly positive AT, strongly positive SN and strongly positive PBC (≥18). Dependent variables were constructed for the intentions by classifying employees into low intenders (Likert score ≤0) versus high intenders (Likert score >0) and used as dependent variable in logistic regression models (one model for each intention). As is commonly done in TBC studies, the full models were presented.

The effects of socio-demographic factors were analyzed by using logistic regression models with TPB factors (AT, SN and PBC) as dependent variables. First univariable analyses were done; for variables significant (P<0.05) in the univariable analyses, correlations between pairs of variables were evaluated using the Spearman Rank correlations. If two variables had a correlation coefficient of ≥|0.6|, only one of the variables was included in the further multivariable analysis. Marital status and having a child were correlated. Having a child was selected for the multivariable analysis because of higher statistical significance. Multivariable analyses were tested using backward reduction. Variables in multivariable models with P < 0.05 were retained. All two-way interactions between variables in the final multivariable models were tested, but no significant interactions were found. Confounding was checked during the model building process by evaluating the change in the beta estimate of other variables when a variable was removed from the models. If this change in beta estimate was >30%, the variable was considered a confounder. The analyses were done by statistical software Statistical Package for Social Scientists (SPSS) and Stata release 14 (StataCorp LLC, USA).

## Results

### Descriptive statistics

**Fig 2:**
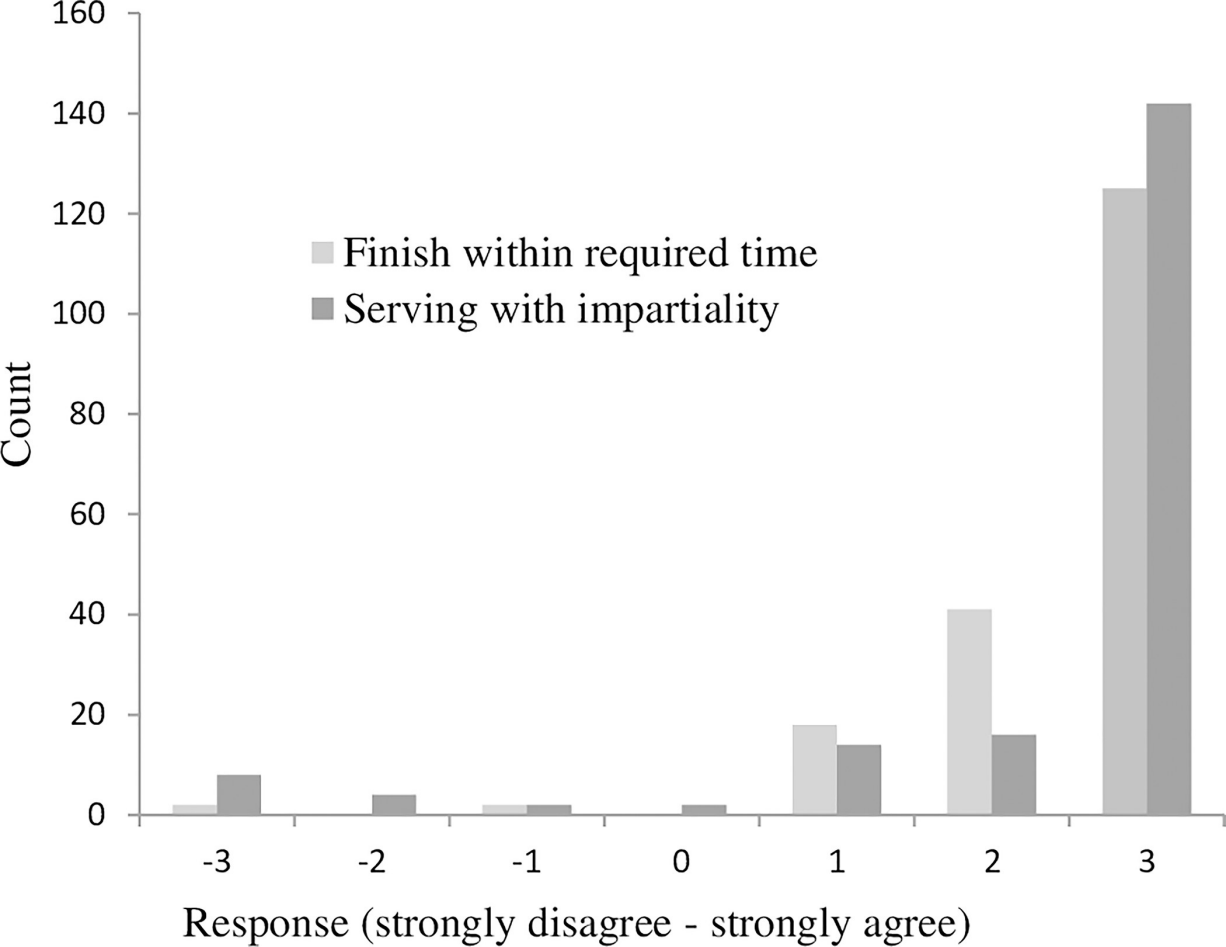
Employees’ intentions of public service performance towards customers’ satisfaction.

Majority of employees had a positive intention to deliver public service towards customers’ satisfaction. In general, a larger number of employees had positive intention to perform their job towards customers’ satisfaction. However, a larger number of employees had a positive intention to serve their customers with impartiality (88.8%) than serving their customers to finish customers’ issues within the required time (66.5%) (Fig 2).

Employees had a positive AT to serve their customers with impartiality (98%) and to finish customers’ issues within the required time (97%). Similarly employees had a positive PBC to serve their customers with impartiality and to finish customers’ issues within the required time. Unlike in case of AT and PBC, the percentage of employees for SN was very low both to serve customers with impartiality and to finish customers issues within the required time. Descriptive statistics of the TPB factors is presented in Table 2.

**Table 2:** Descriptive statistics of attitude (AT), subjective norms (SN) and perceived behavioral control (PBC) measured with respect to the intentions towards satisfying customers.

Service delivery	Variable	Cronbach’s alpha	Weak (n)	Moderate (n)	Strong positive (n)	Positive responses (n (%))
**Finish customers’ issues within required time**	AT (bb*oe)	0.94	4	29	155	184 (98)
	SN (nb*mc)	0.86	112	62	14	76 (40)
	PBC1 (cb*ppc)	0.35	11	155	22	177 (94)
	PBC2 (cb*ppc)		14	51	123	174 (93)
**Serving customers with impartiality**	AT	0.91	6	92	90	182 (97)
	SN	0.95	107	63	18	81 (43)
	PBC1 (cb*ppc)	0.37	34	138	16	154 (82)
	PBC2 (cb*ppc)		20	34	134	168 (89)

AT = Attitude, bb = behavioral beliefs, oe = outcome evaluation, SN = subjective norm, nb = normative belief, mc = motivation to comply, PBC = perceived behavioral control, cb = control belief, ppc = perceived power of control, 1and 2 = the 1^st^ and the 2nd items that had Cronbach’s alpha value <0.7 for perceived behavioral control in both intentions and were used as separate variables without averaging them.

On average, employees that participated in the study had 13 years of public service experience, 7935 Ethiopian Birr average salary, and were 35 years of age. Majority of employees had first degree level of education (69%), had no experience of working as a leader (69%) and trained about improving civil service delivery (56%). The detail of socio-demography of participant employees is summarized (Table 3).

**Table 3:** Socio-demographic factors expected to influence attitude, subjective norms and perceived behavioral control of employees’ that are associated with their intention.

Socio-demographic factor	Level	Number	Proportion
**Gender**	Male	121	64
	Female	67	36
**Level of education**	Diploma	27	14
	First degree	130	69
	Master’s degree	31	16
**Age**	≤ 30 years	49	26
	31 to 40 years	113	60
	>40 years	26	14
**Marital status**	Single	63	34
	Couple	115	61
	Divorced & widow	10	5
**Family size**	≤ 3 persons	95	51
	4 to 6 persons	78	41
	> 7 persons	15	8
**Having child**	No	87	46
	Yes	101	54
**Salary**	≤ 7,000	48	26
	> 7,000 to 10,000	93	49
	> 10,000	47	25
**Presence of income other than salary**	No	166	88
	Yes	22	12
**Years of service**	< 10 years	67	36
	10 to 15 years	68	36
	> 15 years	53	28
**Experience of leadership**	No	130	69
	Yes	58	31
**Manager currently**	No	164	87
	Yes	24	13
**Training about improving civil service delivery**	No	83	44
	Yes	105	56

### TPB factors associated with intentions to perform activities towards customers’ satisfaction

The product composites of behavioral belief statements and the corresponding outcome evaluations, and normative beliefs and motivation to comply were highly correlated (Cronbach’s alpha ≥0.7) in both the intentions, suggesting that they address the same construct (had internal consistency). Therefore, the product composites were averaged to obtain one single measure (mean score) for TPB factors. The product composites for control belief statements and the corresponding perceived power of control did not have internal consistency, did not address the same construct. Therefore, product composites were used as separate TPB factors.

Employees’ ATs were significantly and positively associated with the intention to serve customers with impartiality. Subjective norm were significantly associated with the intention to finish customers’ issues within required time (Table 4).

**Table 4:** A summary of the association (P<0.05) between employees’ attitude, subjective norms and perceived behavioral control with their intention.

Public service	Variable	Level	OR^1^ (95% CI^2^)	P-value
**Finishing customers’ issues within required time**	AT (bb*oe)	Weak	Ref^3^.	
		Moderate	1.02 (0.06–17.19)	0.98
		Strong positive	14.80 (0.89–246.15)	0.06
	SN (nb*mc)	Weak	Ref.	
		Moderate	0.24 (0.11–0.54)	0.01
		Strong positive	0.26 (0.07–0.98)	0.04
	PBC1 (cb*ppc)	Weak	Ref.	
		Moderate	0.16 (0.02–1.09)	0.06
		Strong positive	0.13 (0.01–1.12)	0.06
	PBC2 (cb*ppc)	Weak	Ref.	
		Moderate	1.16 (0.22–5.94)	0.86
		Strong positive	2.57 (0.53–12.53)	0.24
**Serving customers with impartiality**	AT (bb*oe)	Weak	Ref.	
		Moderate	18.42 (1.89–179.83)	0.01
		Strong positive	146 (10.54–2035.58)	0.01
	SN (nb*mc)	Moderate	Ref.	
		Strong positive	1.5 (0.52–4.33)	0.45
	PBC1 (cb*ppc)	Moderate	Ref.	
		Strong positive	1.37 (0.35–5.34)	0.65
	PBC2 (cb*ppc)	Weak	Ref.	
		Moderate	0.57 (0.11–3.01)	0.51
		Strong positive	1.34 (0.28–6.37)	0.71

^1^Odds ratio

^2^95% confidence interval

^3^Reference category, AT = Attitude, bb = behavioral beliefs, oe = outcome evaluation, SN = subjective norm, nb = normative belief, mc = motivation to comply, PBC = perceived behavioral control, cb = control belief, ppc = perceived power of control, 1and 2 = the 1^st^ and the 2nd items that had Cronbach’s alpha value <0.7 for perceived behavioral control in both intentions and were used as separate variables without averaging them.

### Socio-demographic factors associated with TPB factors

Of the 12 socio-demographic factors evaluated, five were associated with SN in the univariable analysis (Table 5). Attitude and PBC had no association with any of the socio-demographic factors.

**Table 5:** A summary of univariable analysis of socio-demographic factors significantly associated (P<0.05) with employees’ theory of planned behavior factors to finish customers’ issues within required time.

Socio-demographic factor	Level	OR^1^ (95% CI^2^)	P-value
**Gender**	Male	Ref^3^.	
	Female	1.32 (0.72–24.20)	0.37
**Level of education**	Diploma	Ref.	
	First degree	0.40 (0.17–0.94)	0.03
	Master’s degree	0.43 (0.15–1.25)	0.12
**Age**	≤ 30 years	Ref.	
	31 to 40 years	0.69 (0.35–1.37)	0.29
	>40 years	0.71 (0.27–1.86)	0.48
**Marital status**	Single	Ref.	
	Couple	0.38 (0.20–0.71)	0.01
	Divorced & widow	0.53 (0.14–2.08)	0.36
**Family size**	≤ 3 persons	Ref.	
	4 to 6 persons	0.68 (0.34–1.75)	0.27
	> 7 persons	1.05 (0.49–2.22)	0.90
**Having child**	No	Ref.	
	Yes	0.41 (0.23–0.75)	0.01
**Salary**	≤ 7,000	Ref.	
	> 7,000 to 10,000	0.47 (0.23–0.95)	0.04
	> 10,000	0.33 (0.14–0.77)	0.01
**Presence of income other than salary**	No	Ref.	
	Yes	0.82 (0.33–2.07)	0.68
**Years of service**	< 10 years	Ref.	
	10 to 15 years	0.77 (0.39–1.51)	0.44
	> 15 years	0.47 (0.22–1.01)	0.05
**Experience of leadership**	No	Ref.	
	Yes	0.62 (0.33–1.19)	0.15
**Manager currently**	No	Ref.	
	Yes	1.56 (0.66–3.69)	0.31
**Training about improving civil service delivery**	No	Ref.	
	Yes	0.51 (0.28–0.93)	0.03

^1^Odds ratio

^2^95% confidence interval

^3^Reference category

Of the five independent variables significantly associated with subjective norm, level of education, having a child and training about improving civil service delivery were remained in the final multivariable model. The detail of association in multivariable analysis is presented (Table *6*). All the data in this paper are available in supporting information file (S2 Table).

**Table 6:** Socio-demographic factors associated (P<0.05) with subjective norms of employees’ intention to finish customers’ issues within required time.

Socio-demographic factor	Level	OR^1^ (95%CI^2^)	P-value
**Level of education**	Diploma	Ref^3^.	
	First degree	0.37 (0.15–0.91)	0.03
	Master’s degree	0.45 (0.15–1.34)	0.15
**Having child**	No	Ref.	
	Yes	0.42 (0.23–0.78)	0.01
**Training about improving civil service delivery**	No	Ref.	
	Yes	0.53 (0.29–0.98)	0.04

^1^Odds ratio

^2^95% confidence interval

^3^Reference category

## Discussion

### Theoretical implications

This research was undertaken to gain an understanding of the factors influencing employees’ intentions to provide public service that enhance customers’ satisfaction. Additionally, it aims to identify the socio-demographic characteristics linked to employees’ AT, SN and PBC in the context of delivering public services aimed at customer satisfaction. The study was done to explore intentions towards serving customers with impartiality and finishing customers’ issues within the required time. According to the TPB, behavior can be predicted at high accuracy by using behavioral intention as a proxy measure of an action [34]. The theory assumes rational behavior and the intention of a person to perform a certain behavior is assumed to be influenced by their AT, SN and PBC [35]. If we take finishing customers issues within the required time as an example, the actual finishing customers’ issues within the required time is a behavior whereas intention is readiness to finish customers’ issues within the required time [45]. Attitude refers to whether a civil servant is in favor of finishing customers’ issues within the required time, SN is civil servant’s perception of social pressure to finish customers’ issues within the required time and PBC represents the sense of self-efficacy or ability to finish customers’ issues within the required time.

### Intentions of employees to perform activities to satisfy customers

In this study, intentions towards serving customers with impartiality and finishing customers’ issues within the required time were compared. We found that the majority of employees had the intention to implement the suggested public services, but the intention to practice serving customers with impartiality is greater than the intention to serve customers by finishing their issues within the required time. Apparently, serving customers with impartiality is an intangible measure; so employees have a strong intention to serve customers with impartiality, but the intention decreased when the measures become more tangible, finishing customers’ issues within the required time. The reason for this could be a perceived lack of ease of implementation, because of the time it takes [46]. This reflects the high intention of the employees to do something about customers’ satisfaction. By then asking about more specific measure, we could identified that employees intend to work less to finish customers’ issues within the required time, possibly because many of the employees perceived low behavioral control towards finishing customers’ issues within the required time.

### Theory of planned behavior factors

Majority of the employees had high positive AT and high positive PBC towards both intentions. Attitude questions were refereeing to the impact and importance of those public service delivery measures. Therefore, our finding shows that employees recognize both the impact and importance of high level of public service delivery. The PBC was evaluated using statements referring to the ability (skill) and availability of time to implement the public service delivery measures. On average, 78.5% of the employees reported they have the ability and time to implement the stated service delivery measures. The percentage of employees that had a positive SN was low. This means, employees may not be influenced to enact normative beliefs of referent people, for instance by wife in couples. In the area where the study was conducted, no more than half of husbands participate their wives in decision making [47]. Our finding supports this report as most of the respondents (64%) in the current study were males. A husband who do not participate his wife in decision making is less likely to follow her as referent person.

### Associations between TPB factors and intentions

Employees who had a positive AT had higher odds of intention to serve customers with impartiality. We mentioned that, AT was evaluated by questions refereeing to the impact and importance of those public service activities. Similarly, employees who had a positive AT had higher odds of intention to finish customers’ issues within required time. Attitudes for the intention to finish customers’ issues within required time were evaluated by questions referring to the benefits customers expected to have if the civil servant would finish customers’ issues within required time. Employees who had evidence of effectiveness of finishing customers’ issues within required time were motivated to implement this measure.

As the percentage of employees SN was very low in both intentions (serving customers with impartiality and finishing customers issues within the required time), it is not surprising employees’ SN had lower odds of intention towards finishing customers’ issues within required time. In a comparable study on behavior of breaking of posted speed limits Conner et al. [48] found a negative subjective norm not to speed. A reason for the negative association between SN and intention to finish customers’ issues within required time may be due to the fact that many of the employees were trained about improving civil service delivery (56%) and have first degree level of education (69%), those employees may have sufficient knowledge to develop their own beliefs. Therefore, employees respond negatively to the statement about their intention to finish customers’ issues within required time and also have a negative SN. There could not be any influence on employees’ intention whether the referents approve or not approve finishing customer’ issues within required time is important in improving customers’ satisfaction.

### Socio-demographic characteristics associated with TPB factors

Numerous studies have demonstrated that social influences have major impacts on individuals’ behaviors through groups and their social environments including family and school [49, 50]. The influential psychosocial environment is clearly reflected in most citizenship definitions. Community norms, what is expected of the public as ‘good’ is defined as a shared set of expectations about the individual’s role. These norms tell individuals what is expected of them, and what they expect of themselves [51]. As highlighted in theory of reasoned action as subjective norm construct, involving important persons of potential influence may help behavioral changes in the target audience [52]. In the current study, employees who had first degree and master’s level of education and those who have training about civil service delivery had lower odds of SN with regard to the intention to finish customers’ issues within required time. This fact has also been shown in disease control intention of dairy farmers; dairy farmers who had *>*8 grade level of education had lower odds of SN with regard to foremilk stripping [44]. The explanation for this is likely to be employees who have had more education and training about civil service delivery feel more confident to perform public service to satisfy customers by themselves and be less susceptible to the advice of referents.

Having child reduced SN of the intention to finish customers’ issues within required time. It is unlike other studies reported elsewhere that friends and family members’ influences are significant antecedents of subjective norm. For instance friends and family members influence and positively related to subjective norms on purchase intention in the context of halal personal care products [53]. This can be explained in relation to the current life situation in Ethiopia; compared to other employees, employees are least paid. Most employees are unable to cover the costs of their child care and other related costs. Because of this reason most women bring their child with them and do their office responsibility while giving care to their child; their time is shared to care their children and to handle customers’ issues. Most of their salary is utilized for purchasing of food items. This shows that although employees belief how other people, who may be in some way important to them would like them to behave (normative beliefs) and the motivation to comply with these referents (motivation to comply), they may not enact to finish customers’ issues within required time.

### Practical implications

Although the intention to serve customers with impartiality is greater than that of finishing customers’ issues within the required time, our finding had shown that majority of employees had the intention to implement the suggested public services. That means if other things are which are necessary in serving customers are fulfilled there is a possibility that employees perform their work to satisfy customers. Majority of the employees had high positive AT towards both intentions and AT was significantly associated with both intentions. These had shown that employees recognize both the impact and importance of high level of public service delivery. These positive associations indicate that information directed at improving employees’ understanding of the impact of those public service delivery activity will likely increase practicing civil service delivery measures.

Our findings show that interventions to improve customers’ satisfaction should be targeted at AT and SN rather than at PBC. Training and promotion on civil service delivery to employees themselves, to their spouse and friends as well as informing their boss about their influence on changing employees’ intention towards customers’ satisfaction are important. In addition, employees often identify ‘other employees’ as source of ideas to change their behavior. Furthermore, employees are strongly influenced by the perception whether a given measure is actually applicable on the civil service delivery. Therefore, forming employees groups as an opportunity for exchanging ideas and accessing advice and practical demonstration of civil service delivery measures may also be important to improve customers’ satisfaction.

We tested for associations between 12 socio-demographic factors and the 3 TPB factors (AT, SN and PBC) for each of the two public service delivery measures. Large number of comparisons made in this way, results in a high chance of making type I errors. Still, the actual number of significant associations was only three, suggesting that social and demographic factors were hardly linked to the TPB factors. We discussed the factors that showed statistically significant associations, but for all of these, we should mention the fact that they may well have resulted from chance and have no biological interpretation.

## Conclusions

The present study explores the intention of employees and identified determinants of employees’ motivation towards customers’ satisfaction. The majority of employees has a positive intention, and has a positive AT towards customers’ satisfaction. However, SN for finishing customers’ issues within required time was low. Attitude was positively associated with the intention to serve customers with impartiality. Subjective norm was negatively associated with the intention to finish customers’ issues within required time. Therefore, to increase the intention of employees’ public service delivery hence customers’ satisfaction, interventions should be directed at changing employees’ AT and their SN.

## Supporting information

S1 Table English version of the whole questionnaire. (DOCX)

S2 Table All the data used in the paper. (XLSX)
