# Supplementary material for: Explaining motivational factors of employees’ behavior towards customers’ satisfaction using the theory of planned behavior
Source: PLoS One. 2024 Nov 22;19(11):e0314431. doi: 10.1371/journal.pone.0314431 (PMC11584082; doi:10.1371/journal.pone.0314431)
Supplement: S1 Table — (DOCX) [file pone.0314431.s001.docx]

**Questionnaire to study motivational factors of employees’ behavior towards customers’ satisfaction**

Dear respondent,

The purpose of this questionnaire is to obtain data for the research “Explaining motivational factors of employees’ behavior towards customers’ satisfaction using the Theory of Planned Behavior”. The goal of the study is to investigate factors motivating employees in public organizations towards customers’ satisfaction. Your response is important for the success of the study. So, I kindly request you to complete and turn back the questionnaire within one week. Your response will be confidential; I assure you that the data will be analyzed and reported anonymously.

Please be aware that you have the right to stop or withdraw from the study with any reason.

Thank you in advance for your willingness to complete the questionnaire.

Respondent number: **__________**

**Demographic profile**:

1. Gender: Male Female
2. Level of education__________________________________
3. Religion __________________________
4. Age _____________________________
5. Marital status Single Married Divorced widowed
6. Family size ______________
7. Having child No Yes
8. Salary ________________
9. Presence of income other than salary No Yes
10. Leadership style _____________________________
11. Years of service ________________
12. Experience of leadership No Yes
13. Are you manager currently No Yes
14. Membership to political party No Yes
15. Training about improving civil service delivery No Yes

**Intention:** Finishing customers’ issues within required time

| In the near future I plan to improve finishing customers’ issues within required time to satisfy customers in the public service delivery | Strongly disagree -3 -2 -1 0 1 2 3 Strongly agree |
| --- | --- |

**Attitude for** finishing customers’ issues within required time

| **Behavioural beliefs** |  |
| --- | --- |
| Finishing customers’ issue within required time increases their satisfaction | Strongly disagree 1 2 3 4 5 6 7 Strongly agree |
| Finishing customers’ issue within required time save customers’ time | Strongly disagree 1 2 3 4 5 6 7 Strongly agree |
| Finishing customers’ issue within required time reduce customers’ energy | Strongly disagree 1 2 3 4 5 6 7 Strongly agree |
| Finishing customers’ issue within required time save customers’ money | Strongly disagree 1 2 3 4 5 6 7 Strongly agree |
| **Outcome evaluation** |  |
| Finishing customers’ issues within required time is important to increase their satisfaction | Strongly disagree -3 -2 -1 0 1 2 3 Strongly agree |
| Saving customers’ time by finishing customers’ issues within required time is important to increase customers’ satisfaction | Strongly disagree -3 -2 -1 0 1 2 3 Strongly agree |
| Reducing customers’ tiredness is important to increase customers’ satisfaction | Strongly disagree -3 -2 -1 0 1 2 3 Strongly agree |
| Saving customers money by finishing customers’ issue within required time increase customers’ satisfaction | Strongly disagree -3 -2 -1 0 1 2 3 Strongly agree |

**Subjective Norm for** finishing customers’ issues within required time

| **Strength of normative belief** |  |
| --- | --- |
| What, according to your knowledge, is the opinion of the following people regarding customers‘ satisfaction in public service delivery |  |
| My spouse think that finishing customers’ issues within required time is | Very unimportant 1 2 3 4 5 6 7 Very important |
| My boss think that finishing customers’ issues within required time is | Very unimportant 1 2 3 4 5 6 7 Very important |
| My friends think that finishing customers’ issues within required time is | Very unimportant 1 2 3 4 5 6 7 Very important |
| Other civil servants think that finishing customers’ issues within required time is | Very unimportant 1 2 3 4 5 6 7 Very important |
| **Motivation to comply** |  |
| Does the opinion of the following people regarding finishing customers’ issue within required time influence your intention to improve finishing customers’ issues within required time? |  |
| Spouse | Not at all -3 -2 -1 0 1 2 3 Very much |
| Boss | Not at all -3 -2 -1 0 1 2 3 Very much |
| Friends | Not at all -3 -2 -1 0 1 2 3 Very much |
| Other civil servants | Not at all -3 -2 -1 0 1 2 3 Very much |

**Perceived Behavioural Control for** finishing customers’ issues within required time

| **Strength of Control Beliefs** |  |
| --- | --- |
| Finishing customers’ issues within required time is difficult | Strongly disagree 1 2 3 4 5 6 7 Strongly agree |
| Finishing customers’ issues within required time is time consuming | Strongly disagree 1 2 3 4 5 6 7 Strongly agree |
| **Power of Factors to Influence the Behaviour** |  |
| I know how to finish customers’ issues within required time | Strongly disagree -3 -2 -1 0 1 2 3 Strongly agree |
| I have time to finish customers’ issues within required time | Strongly disagree -3 -2 -1 0 1 2 3 Strongly agree |

**Intention:** Working with impartiality

| In the near future I plan to improve working with impartiality to satisfy customers in the public service delivery | Strongly disagree -3 -2 -1 0 1 2 3 Strongly agree |
| --- | --- |

**Attitude for** working with impartiality

| **Behavioral beliefs** |  |
| --- | --- |
| Serving with impartiality increases satisfaction of any tribe/nationalities | Strongly disagree 1 2 3 4 5 6 7 Strongly agree |
| Serving with impartiality decreases serving based on political power | Strongly disagree 1 2 3 4 5 6 7 Strongly agree |
| Serving with impartiality decreases serving based on previous knowledge | Strongly disagree 1 2 3 4 5 6 7 Strongly agree |
| Serving with impartiality decreases serving based on relativeness | Strongly disagree 1 2 3 4 5 6 7 Strongly agree |
| Serving with impartiality decreases serving based on economic status | Strongly disagree 1 2 3 4 5 6 7 Strongly agree |
| Serving with impartiality increases satisfaction of customers of any religion | Strongly disagree 1 2 3 4 5 6 7 Strongly agree |
| **Outcome evaluation** |  |
| Serving customers without considering their tribe/nationality increases their satisfaction | Strongly disagree -3 -2 -1 0 1 2 3 Strongly agree |
| Serving customers without considering their leadership power increases customers satisfaction | Strongly disagree -3 -2 -1 0 1 2 3 Strongly agree |
| Serving customers without considering previous knowledge increases their satisfaction | Strongly disagree -3 -2 -1 0 1 2 3 Strongly agree |
| Serving customers without considering their relativeness increases their satisfaction | Strongly disagree -3 -2 -1 0 1 2 3 Strongly agree |
| Serving customers without considering their economic status increases their satisfaction | Strongly disagree -3 -2 -1 0 1 2 3 Strongly agree |
| Serving customers without considering their religion increases their satisfaction | Strongly disagree -3 -2 -1 0 1 2 3 Strongly agree |

**Subjective Norm for** working with impartiality

| **Strength of normative beliefs** |  |
| --- | --- |
| What, according to your knowledge, is the opinion of the following people regarding customers‘ satisfaction in public service delivery |  |
| Spouse | Strongly disagree 1 2 3 4 5 6 7 Strongly agree |
| Boss | Strongly disagree 1 2 3 4 5 6 7 Strongly agree |
| Friends | Strongly disagree 1 2 3 4 5 6 7 Strongly agree |
| Other civil servants | Strongly disagree 1 2 3 4 5 6 7 Strongly agree |
| **Motivation to comply** |  |
| Does the opinion of the following people regarding working with impartiality influence your intention to improve working with impartiality? |  |
| Spouse | Not at all -3 -2 -1 0 1 2 3 Very much |
| Boss | Not at all -3 -2 -1 0 1 2 3 Very much |
| Friends | Not at all -3 -2 -1 0 1 2 3 Very much |
| Other civil servants | Not at all -3 -2 -1 0 1 2 3 Very much |

**Perceived Behavioural Control for** working with impartiality

| **Strength of Control Beliefs** |  |
| --- | --- |
| Working with impartiality is difficult | Strongly disagree 1 2 3 4 5 6 7 Strongly agree |
| Working with impartiality is time consuming | Strongly disagree 1 2 3 4 5 6 7 Strongly agree |
| Working with impartiality is expensive | Strongly disagree 1 2 3 4 5 6 7 Strongly agree |
| **Power of Factors to Influence the Behaviour** |  |
| I know how to work with impartiality | Strongly disagree -3 -2 -1 0 1 2 3 Strongly agree |
| I have time to work with impartiality | Strongly disagree -3 -2 -1 0 1 2 3 Strongly agree |
| I can afford to to work with impartiality | Strongly disagree -3 -2 -1 0 1 2 3 Strongly agree |
